# Dentin phosphophoryn in the matrix activates AKT and mTOR signaling pathway to promote preodontoblast survival and differentiation

**DOI:** 10.3389/fphys.2015.00221

**Published:** 2015-08-07

**Authors:** Asha Eapen, Anne George

**Affiliations:** Brodie Tooth Development Genetics and Regenerative Medicine, Department of Oral Biology, University of Illinois at ChicagoChicago, IL, USA

**Keywords:** dentin phosphophoryn, cell proliferation, pereodontoblasts, signaling, odontoblasts, differentiation

## Abstract

Dentin phosphophoryn (DPP) is an extracellular matrix protein synthesized by odontoblasts. It is highly acidic and the phosphorylated protein possesses a strong affinity for calcium ions. Therefore, DPP in the extracellular matrix can promote hydroxyapatite nucleation and can regulate the size of the growing crystal. Besides its calcium binding property, DPP can initiate signaling functions from the ECM (Extracellular matrix). The signals that promote the cytodifferentiation of preodontoblasts to fully functional odontoblasts are not known. In this study, we demonstrate that preodontoblasts on a DPP matrix, generates mechanical and biochemical signals. This is initiated by the ligation of the integrins with the RGD containing DPP. The downstream biochemical response observed is the activation of the AKT(protein kinase B) and mTOR (mammalian target of rapamycin) signaling pathways leading to the activation of the transcription factor NF-κB (Nuclear factor κB). Terminal differentiation of the preodontoblasts was assessed by identifying phosphate and calcium deposits in the matrix using von Kossa and Alizarin red staining respectively. Identifying the signaling pathways initiated by DPP in the dentin matrix would help in devising strategies for dentin tissue engineering.

## Introduction

Odontoblasts are post-mitotic neural crest-derived mesenchymal cells that are responsible for the production of dentin (Hao et al., 2004). The major acidic NCPs (non-collagenous proteins) are the dentin phosphophoryn (DPP) and the dentin sialoprotein (DSP). The parent protein DSPP has been reported to be a compound protein that encodes DSP at the amino end and DPP at the carboxyl end. Earlier work by Stetler-Stevenson and Veis (1983) and Butler (1998) has shown based on protein isolation and amino acid sequencing the inherent differences that exist between DPP and DSP (Butler, 1998; Butler et al., 2002). Recently, we provided evidence to demonstrate that DSPP (Dentin sialophosphoprotein) gene contains an internal ribosome entry site (IRES) that directs the synthesis of DPP (Zhang et al., 2013). This mechanism should account for unequal amounts of DPP and DSP.

Apart from its role in mineralization, DPP has also been shown to actively participate in cell signaling events leading to differentiation of precursor mesenchymal cells. DPP contains a conserved integrin-binding RGD domain. We and others have shown that binding of DPP to integrins on the cell surface could activate intracellular signals (Jadlowiec et al., 2004, 2006; Eapen et al., 2012a,b). These signals when translocated to the nucleus altered gene functions in undifferentiated mesenchymal cells.

DPP is predominantly present at the mineralization front of the developing dentin matrix (Hao et al., 2004, 2009; Suzuki et al., 2009). However, the signaling response of preodontoblasts to DPP in the ECM and the functional readout has not been investigated yet. The objective of this study was to investigate the signaling function of DPP in the matrix by which T4-4 (Hao et al., 2002) preodontoblast cells promote cell proliferation and cell survival.

## Materials and methods

### Cell culture

Rat immortalized pre-odontoblast cells (T4-4) (Hao et al., 2002) were cultured in DMEM/F-12 medium (growth media) supplemented with 10% FBS and 1% penicillin-streptomycin. Twelve to sixteen hours before the start of the experiment, the cells were cultured in growth medium supplemented with 1% FBS. Non-tissue culture grade six well plates were coated with recombinant DPP (750 ng/ml) in carbonate buffer. The cells were rinsed with PBS, trypsinized, and seeded at 80% confluency. T4-4 cells seeded on 6 well plates coated with carbonate buffer served as control (Eapen et al., 2012a).

### DPP coating on non tissue culture plates

Non tissue culture plates coated with DPP were prepared by soaking the plate with 750 ng/ml of DPP as described earlier (Eapen et al., 2012a).

### Quantitative real time PCR

RNA was extracted according to the manufacturer's recommended protocol by using Trizol (Invitrogen). RT-qPCR was performed with DNase I (Promega) treated RNA. A total of 1 μg of total RNA was reverse transcribed for 90 min at 50°C with Superscript III (GIBCO) (Eapen et al., 2012a). After RNA extraction, quantitative real-time PCR (qPCR) analysis were carried out using ABI Step One Plus machine. Expression for Cyclin A1, Cyclin D1, Cyclin B1, CDK4, and GAPDH transcripts were analyzed by qPCR during its linear phase. The relative gene expression level was estimated by using the $2_{\text{T}}^{-\Delta \Delta C}$ method where C_T_ value = log-linear plot of PCR signal versus the cycle number. ΔC_T_ = C_T_ value of target gene-C_T_ value of GAPDH (Eapen et al., 2012a). Primers were obtained from Qiagen.

### Identification of integrins

RNA was extracted from T4-4 cells seeded on DPP coated plates at 4 and 24 h time points. The samples were then subjected to genomic DNA contamination removal using elimination mixture (Qiagen), and first strand cDNA synthesis was carried out using the RT^2^ Easy First Strand Kit (Qiagen) as described by the manufacturer's protocol. PCR array analysis was performed on the predesigned Focal Adhesion Profiler™ array of 96 genes (Super Array Bioscience Corporation). RT-qPCR was performed on ABI Step One Plus machine. For data analysis, the ΔΔCt method was used; for each gene fold-changes were calculated as difference in gene expression between untreated controls and treated cell cultures. The result from the array was interpreted using software provided by Qiagen.

### Identification of cell surface receptor

Integrin receptors from T4-4 cells were isolated as published earlier (Eapen et al., 2012a). Briefly, the cell membrane proteins were extracted after treating T4-4 cells with GST-DPP. The membrane proteins were eluted with glutathione, lyophilized and western blotting performed with anti-α4 and anti-β1 antibody (1:500).

### Cell cycle analysis by flow cytometry

The effect of DPP on the different phases of the cell cycle in T4-4 cells was determined by flow cytometry. Cells were plated on DPP substrate for 24 h, harvested and fixed in ice cold 70% ethanol. The pellet was washed in ice-cold PBS followed by staining with propidium iodide (PI) at room temperature for 40 min. Cells were then rinsed with PBS and the cell cycle profiles were analyzed by flow cytometry using CellQuest software used for cell cycle analysis.

### Immunofluorescence

T4-4 cells were cultured on rDPP coated non-tissue culture grade glass cover slips for various time points. Cells were fixed in 4% paraformaldehyde, then permeabilized with 0.1% Triton X-100 in PBS for 5 min and rinsed twice with wash buffer (Eapen et al., 2012a). After blocking for 30 min, the cells were then incubated overnight in primary antibody namely, Integrin β1 (kind gift from S. Carbonetto, McGill University, Montreal, Quebec), anti-FAK and anti-Paxillin followed by incubation with a Cy3-conjugated secondary antibody for 1 h. The cells were washed three times with PBS and the cover glass was mounted using mounting media (Vector shield, CA) and imaged using confocal microscopy (Zeiss). The cytoskeleton of the cells were stained with actin (1:100) at different time points followed by DAPI staining for the nucleus and visualized with an Axio Observer D1 Fluorescence Microscope (Zeiss, NY) equipped with Axiovision imaging software (Zeiss, NY).

### Western blot analysis

Activation of the focal adhesion components, survival pathways (mTOR, AKT, and NF-κB), cyclins and odontoblast differentiation markers were determined by western blot analysis. Total proteins were extracted from T4-4 cells grown on DPP coated plates with or without mineralization media using M-per reagent (Pierce, IL) (Eapen et al., 2012a). A total of 35 μg of the protein was resolved on a 10% SDS-PAGE gel under reducing conditions. After electrophoresis, the proteins were electro-transferred onto nitrocellulose membrane (Bio-Rad Laboratories, CA), blocked with 3% BSA in 1X PBS, probed with either anti-FAK (1:500) (Santa cruz, CA), anti-paxillin, anti-phospho FAK (1:500) (Santa Cruz, CA), anti-phospho paxillin (1:500) (Cell signaling, MA), anti- phospho AKT(1:500) (Cell signaling, MA), anti-AKT (1:500) (Cell signaling, MA), anti-phospho mTOR (1:500) (Cell signaling, MA), anti-mTOR (1:500) (Cell signaling, MA), anti-phospho NFkB (1:500) (Cell signaling, MA), anti- NF-kB (1:5000) (Chemicon), anti-cyclin D1 (1:500) (Santa Cruz, CA), anti CDK4 (1:500) (Santa Cruz, CA), anti-DMP1 (1:500) anti-DPP (1:500) and anti-DSP (1:2000). HRP-conjugated goat anti-rabbit IgG were used for detection (Chemicon International, CA). Lightening chemiluminescence reagent (Perkin-Elmer Life Sciences, MA) was used as substrate for HRP. Each membrane was then carefully washed, treated for 5 min with a stripping buffer (Pierce, IL), washed with PBS and processed as above with anti- tubulin (1:10,000, Sigma, MO) antibody and HRP conjugated goat anti-mouse IgG (Eapen et al., 2012a).

### *In vitro* assay to determine cell differentiation

Mineralization microenvironment was induced by the addition of 10 mM β-glycerophosphate and 100 μg/ml ascorbic acid (CitySigma–Aldrich., MO) along with 10 nM dexamethasone (CitySigma–Aldrich., MO) in the growth media as published (Eapen et al., 2012a). Total proteins were extracted at 7, 14, and 21 days from T3 cells grown on DPP coated plates (Eapen et al., 2012a).

### von Kossa staining for phosphates in the mineralized nodule

von Kossa staining was performed as published (Eapen et al., 2012a) to determine the presence of phosphate in the mineralized nodules. T4-4 cells were grown to 60% confluency on DPP coated plates and the growth media was then replaced with mineralization media for 7, 14, and 21 days. The cells were fixed in formalin for 20 min, washed twice with distilled water and then stained with 1% silver nitrate solution (Sigma–Aldrich., MO) for 30 min and photographed. Cells grown on DPP coated plates without mineralization media and cells on tissue culture plate with mineralization media served as controls.

### Alizarin red S staining for calcium in the mineralized nodule

T4-4 cells were grown to 60% confluency on DPP coated plates. The growth media was replaced with mineralization media for 7, 14, and 21 days. The cells were fixed in formalin for 20 min at room temperature and washed with distilled water. 2% Alizarin Red solution was added to fixed cells and incubated for 10–20 min (Eapen et al., 2012a). The cells were then rinsed with distilled water and were imaged.

### Statistical analysis

PCR-array analysis was performed for both experimental groups (*n* = 3). Means and standard deviations (SE) were calculated, and the statistical significance of differences among each group was examined by student *t*-test between both groups (*p* < 0.05).

## Results

### DPP mediates cell adhesion

**Figure 1:**
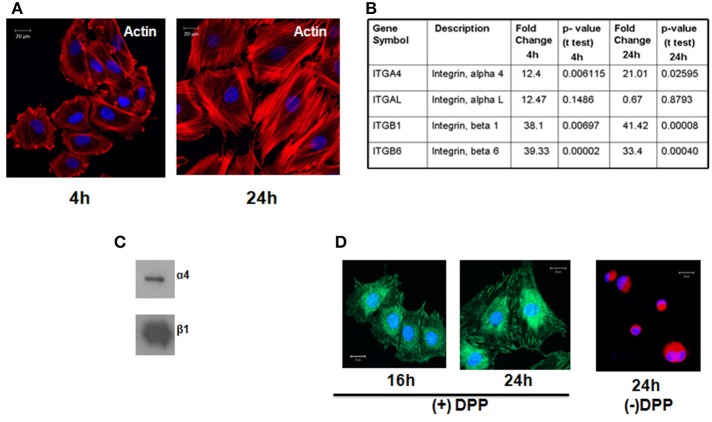
**T4-4 preodontoblasts adheres to DPP substrate by α4β1intergrin and activates the cytoskeletal elements**. **(A)** T4-4 cells were seeded on DPP-coated non-tissue culture cover glass and cultured for 4 and 24 h time point. Immunofluorescences staining with Texas-red phalloidin show accumulation of actin filaments at the membrane surface at 4 h and polarized filaments at 24 h. No cell attachment was observed in the absence of DPP. **(B)** Identification of Integrin receptors. Expression of alpha and beta subunits of integrins present on T4-4 cells at 4 and 24 h when plated on DPP substrate. Total RNA was isolated and Focal Adhesion Profiler™ PCR array analysis was performed at 4 and 24 h on the DPP adherent T4-4 cells. Fold change increase of the integrin receptors is represented in **(B)**. **(C)** Total membrane proteins were isolated from T4-4 cells adhered to DPP substrate and Western Blot analysis was performed with anti-alpha 4 and anti-beta 1 integrin antibodies. **(D)** Confocal imaging show the localization of β1 integrin on the membrane of T4-4 cells adhered to DPP substrate. Cells seeded on plates coated with carbonate buffer (absence of DPP) served as the control.

T4-4 cells were seeded on DPP-coated non-tissue culture cover glass as published before (Eapen et al., 2012a) and cultured for 8 and 24 h. Actin staining was performed on the fixed cells. Confocal microscopy image clearly showed the dynamic nature of the actin cytoskeleton in the cells attached on DPP substrate at 4 and 24 h (Figure 1A). Cells seeded on cover glass without DPP failed to adhere.

### Identification of integrin receptors

In order to identify the integrins specific for the binding of T4-4 cells on DPP coated plates we performed PCR-based integrin pathway-specific super array. RNA was isolated at 4 and 24 h from T4-4 cells that were seeded on the DPP coated substrate and real time super array was performed. Analysis revealed an increase in the expression of integrin αL, α4, β1, and β6 genes from the pathway-specific array containing several alpha and beta integrin receptors in the presence of DPP substrate (Figure 1B). T4-4 cells seeded on tissue culture plates served as controls. Specifically, the expression of integrin αL, α4, β1, and β6 genes were up regulated at 4 h time point respectively. Intracellular proteins responsible for transducing DPP mediated extracellular signals use integrins as a mediator molecule. These results suggest that the preodontoblasts utilize these integrins in DPP mediated cell adhesion and signaling. Interestingly, α4 and β1 was observed to be up-regulated at 4 and 24 h time points suggesting that α4β1 integrin might be involved during early stages of DPP mediated adhesion. Having identified the possible alpha and beta integrin receptors and in order to validate the array data we performed immunoblot analysis with membrane proteins isolated from adherent cells on DPP substrate. Results from the western blot clearly showed an up-regulation of α4 and β1 integrins in the presence of DPP (Figure 1C). Figure 1D shows the expression of β1 integrin at the focal adhesion points of T4-4 cells when plated on DPP substrate at 16 and 24 h. Cells seeded on plates coated with carbonate buffer served as control and failed to spread.

### DPP facilitates formation of focal adhesion complex

**Figure 2:**
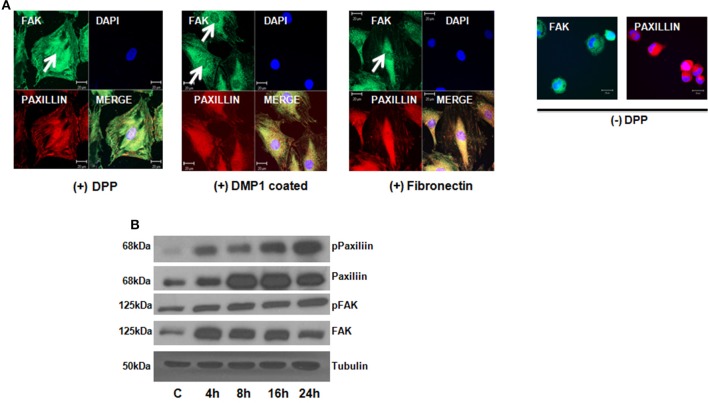
**DPP matrix induces the activation of Focal adhesion complex in T4-4 adhered cells**. **(A)** Immunofluorescence staining of FAK and Paxillin in T4-4 cells cultured on DPP coated non-tissue culture slides for 24 h at 37°C. Nuclear localization of FAK (white arrow) and membrane localization of Paxillin was observed in the DPP mediated T4-4 adherent cells. Absence of nuclear localization of FAK was seen in cells seeded on DMP1 and Fibronectin coated substrates (white arrow) which were positive controls. Cells were rounded in the absence of DPP. Scale bar = 20 μm. **(B)** Western blot analysis of active FAK, total FAK, active Paxillin and total Paxillin showed DPP mediated activation of focal adhesion complexes in T4-4 cells at the specified time points. Experiments were repeated three times and similar results were obtained.

Focal adhesion kinase (FAK) has been shown to regulate integrin mediated survival signaling. We therefore examined if DPP substrate would induce association of FAK and paxillin in preodontoblast cells. Confocal microscope images clearly indicated the co- localization of FAK and paxillin at the focal points on the cell membrane of adherent T4-4 cells at 24 h (Figure 2A). Cells plated on DMP1 and fibronectin substrates were used as positive controls. Interestingly, DPP mediated nuclear translocation of FAK was observed in adherent cells at 24 h time point. Control cells on carbonate buffer coated slides failed to express FAK and Paxillin and were rounded in morphology (Figure 2A). Therefore, we next determined whether protein expression for FAK and Paxillin were up regulated in T4-4 cells in the presence or absence of DPP. Immunoblot analysis confirmed that DPP adherent cells showed significant expression of FAK and Paxillin with increasing time (Figure 2B).

### DPP mediates the activation of AKT-mTOR-NF-kB signaling pathway

**Figure 3:**
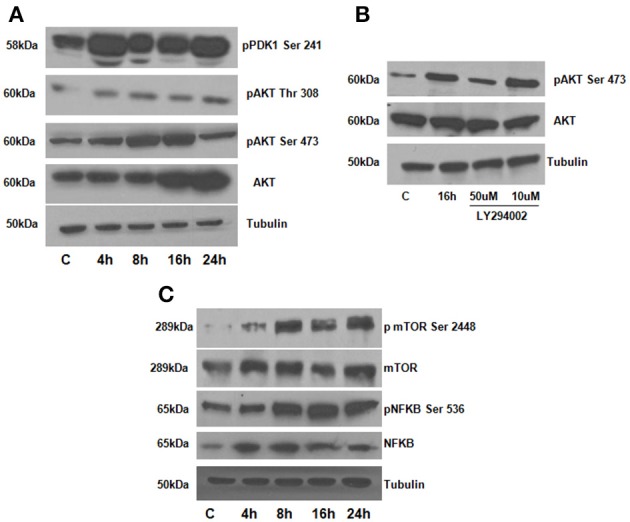
**(A)** DPP activates AKT, mTOR and NFκB signaling pathways in T4-4 cells. T4-4 cells were cultured on DPP coated non tissue culture plates for 4,8,16, and 24 h. Cells were lysed and equal amounts of proteins were separated by SDS-PAGE, transferred to PVDF membrane, and immunoblotted with antibodies against AKT, p-AKT (Ser 473), p-AKT (Thr 308), p-PDK1 (ser 241), and Tubulin **(A)**. **(B)** Inhibitor LY294002 causes in-activation of AKT in DPP mediated adhered T4-4 cells. T4-4 cells were pre-treated with LY294002 10 and 50 μm for 30 min and then seeded on DPP substrate for 16 h. After 16 h, cells were lysed and equal amounts of proteins were separated by SDS-PAGE, transferred to PVDF membrane, and immunoblotted with antibodies against p-AKT, total AKT and tubulin. **(C)** DPP mediated activation of activation of NFκB and mTOR pathways. Total proteins were isolated from the T4-4 adhered cells, lysed and equal amounts of proteins were separated by SDS-PAGE, transferred to PVDF membrane, and immunoblotted with antibodies against p-NFκB, p-mTOR, total NFκB and total mTOR antibodies. Equal loading of the proteins were confirmed by stripping and reprobing the blot with tubulin antibody.

To assess the role of AKT in DPP mediated adhesion, we first determined if adherent T4-4 cells can induce AKT phosphorylation at serine residues (Ser473 and 308), Studies have shown that phosphorylation at Ser 473 is important for the activation of the enzymatic function of AKT kinases (Toker and Newton, 2000). Significant increase in AKT phosphorylation at Ser 473 was observed at 8 and 16 h when compared to control T4-4 cells cultured on tissue culture plates (Figure 3A). AKT phosphorylation at Ser 473 declined at 24 h time point suggesting the role of AKT during early events of cell adhesion and survival. To further corroborate the above results T4-4 cells were pre-treated with LY294002 and then seeded on DPP substrate. LY294002 is a well-known inhibitor of PI3K, which inhibits activation of AKT (Borgatti et al., 2000). As shown in Figure 3B, LY294002 treatment repressed AKT expression in preodontoblasts.

We next investigated if the activation of the AKT pathway by DPP could lead to cell survival by activation of mTOR (mammalian target of rapamycin) and NF-kB activation. mTOR is an important downstream target of AKT signaling pathway leading to its phosphorylation (Harris and Lawrence, 2003; Guertin and Sabatini, 2005; Rosner et al., 2008). To understand whether mTOR was activated by DPP, western blotting analysis was performed on cells plated on the DPP substrate. Results in Figure 3C show an upregulation of phosphorylated mTOR expression from 8 to 24 h time point.

As NF-κB is a transcription factor considered to play a key role in both cell proliferation and apoptosis, therefore, we examined if NF-κB is activated downstream of signaling induced by DPP. In order to validate NF-kB activation, we isolated total proteins from cells seeded on DPP coated plates at 4, 8, 16, and 24 h respectively. A significant increase in the phosphorylated NF-kB (Ser 536) was observed from 8 to 24 h time points respectively (Figure 3C). Taken together, these results strengthened the finding that DPP stimulates the activation of cell adhesion mediated by AKT-mTOR-NF-kB signaling.

### DPP regulates cell cycle controlling proteins

**Figure 4:**
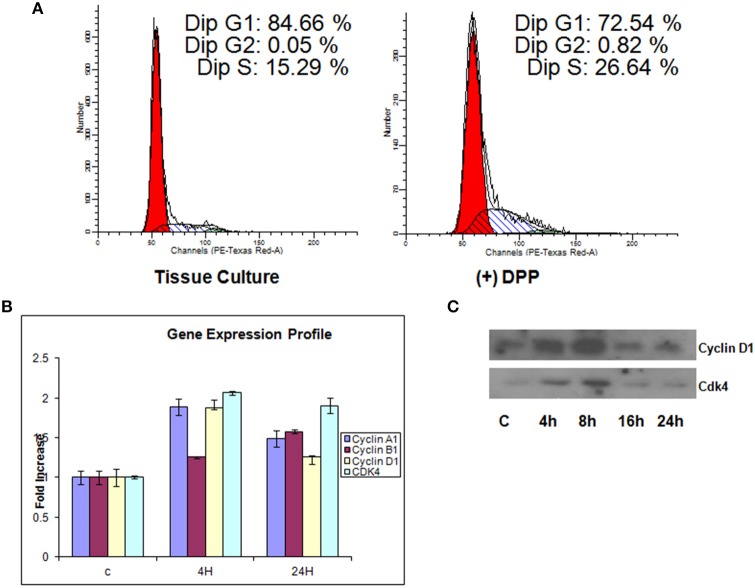
**Illustration of DNA measurement by flow cytometry in cells cultured on tissue culture or DPP coated non-tissue culture plates**. **(A)** Cells were stained by PI solution, and the cell cycle distribution (G0/G1, S, and G2/M) was analyzed by flow cytometry. The distribution and percentage of cells in G_1_, S, and G_2_/M are indicated. T4-4 cells grown on tissue culture plates were used as controls. Flow cytometry analysis showed that 26.6% of cells cultured on DPP coated substrate were seen to be in the S-phase when compared to the cells that were cultured on tissue culture plates. **(B)** Total RNA was isolated from the T4-4 adhered cells at 4 and 24 h and real time PCR was performed for Cyclin A1, Cyclin D1, Cyclin B1, and CDK4. **(C)** Western blot analysis of Cyclin D1, and CDK4 proteins were perfomed with the total proteins isolated from the DPP substrate adhered T4-4 at the specified time points.

We next investigated the regulation of cell-cycle proteins by DPP. For this, T4-4 cells grown on DPP coated plates in the absence of serum for 24 h were harvested and flow cytometric analysis was performed. Cells grown on tissue culture plates were used as control. In contrast to G1 phase growth arrest in differentiated cells, proliferating T4-4 cells stimulated with DPP at 24 h showed a significant increase in the cell population in S phase (Figure 4A). As cell cycle regulation is controlled by a combination of cyclins, cdks, and cdk inhibitors, we therefore examined changes in gene expression for cyclins using Real-Time PCR. Cells seeded on DPP showed significant up regulation of cyclin A1 and D1 and a down regulation of cyclin B1 gene expression at 4 h (Figure 4B). However, Cyclin B1 was upregulated at 24 h. Interestingly, the gene expression level of Cdk4, which is the kinase regulatory partner of cyclin D1, was seen to be up regulated at 4 h when compared with control (Figure 4B). The complex, Cyclin D1 along with its catalytic partner Cdk4 may play a vital role in propelling the cell cycle through the G1/S checkpoint, a critical phase of cell proliferation. We then sought to determine the protein expression levels of cyclin D1 and CDK4 using western blot analysis. As revealed in Figure 4C, DPP significantly up regulated the expression of Cyclin D1 and CDK4 at 4 and 8 h. These results suggest that DPP promotes survival and proliferation of T4-4 cells by activating cyclin D1-CDK4 complex which in turn regulates the G1/S transition.

### DPP promotes terminal differentiation of preodontoblasts

**Figure 5:**
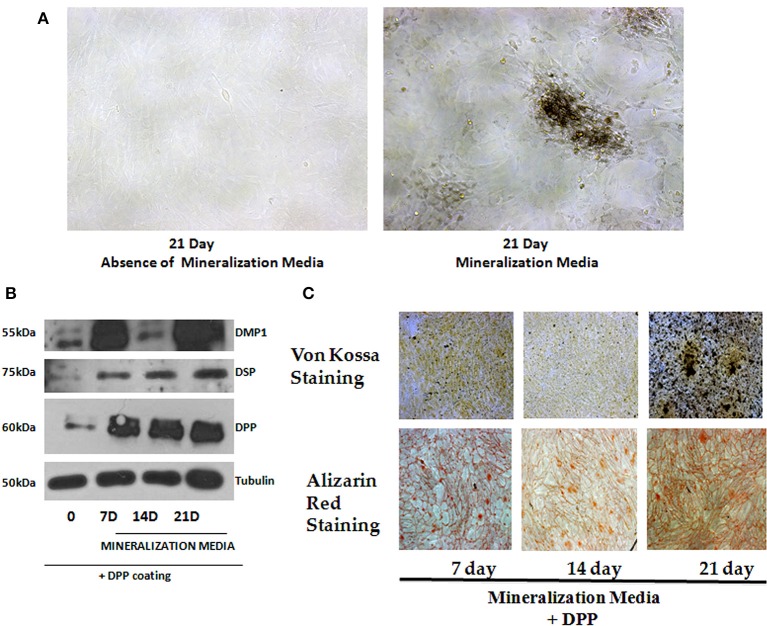
**Role of DPP in terminal differentiation of T4-4 cells**. **(A)** T4-4 cells were cultured on DPP-coated plates in mineralization medium for 7, 14, and 21 days. Light microscopic images of the cells in the presence and absence of mineralization medium at 21 days were taken. Morphological changes in the cells were noticed in the presence of DPP and mineralization medium. **(B)** Total proteins were isolated from the DPP substrate adherent T4-4 cells and grown under mineralization conditions for 7, 14, and 21 days. Immunoblot analysis was performed for DPP, DSP and DMP1. **(C)** Alizarin Red and von Kossa staining were performed on T4-4 cells cultured on DPP substrate for 7, 14, and 21 days, respectively.

As the preodontoblasts differentiate to polarized functional odontoblasts, they undergo a change in cellular morphology with the formation of mineralized matrix. Cells grown on DPP-coated plates were subjected to mineralization for 7, 14, and 21 days, and their morphology was assessed by light microscopy. Distinct morphological changes were observed when cells were cultured in the presence of mineralization medium for 21 days (Figure 5A). Differentiation was assessed by identifying odontoblastic differentiation markers. Data obtained from Western blots showed a significant increase in DSP, DMP1(dentin matrix protein 1) and DPP from day 7 to day 21 when compared with the control cells, which were obtained at 21 days in the absence of differentiation medium (Figure 5B). In order to determine whether DPP could influence the differentiation of preodontoblasts to fully differentiated cells *in vitro*, von-Kossa and Alizarin Red staining was performed at 7, 14, and 21 days time points under mineralization conditions on cells grown on DPP substrate. von Kossa staining showed dark nodules in the extracellular matrix, demonstrating the presence of phosphate in the calcified matrix. Odontogenic terminal differentiation of preodontoblsts was further confirmed by Alizarin Red staining. Results showed staining of the mineralized matrix at 7, 14, and 21 days demonstrating the presence of calcium deposits (Figure 5C).

## Discussion

Mechanisms involved in the terminal differentiation of preodontoblasts to functional odontoblasts are not well-defined. DPP is highly expressed by odontoblasts, however, early on in development expression has been observed in undifferentiated ameloblasts (Begue-Kirn et al., 1998; Bleicher et al., 1999; Paine et al., 2005). Earlier, studies have shown that the intact predentin was unable to promote physiological terminal differentiation of odontoblasts (Karcher-Djuricic et al., 1985). However, non-collagenous fractions from rabbit dentin facilitated the formation of polarized odontoblast-like cells. This suggests that the changing dentin extracellular matrix is not just an inert supporting material but a dynamic vector of information. Therefore, the dentin matrix plays an important role during terminal differentiation of odontoblasts. In this study, we demonstrate that DPP, a major component of the dentin ECM initiates a signaling program that is necessary for the terminal differentiation of preodontoblasts to functional odontoblasts.

We first showed that the preodontoblasts contain α4β1 specific cell surface integrins that can interact with DPP in the matrix. In fact β1 interaction with DPP in the matrix was predominant. Absence of DPP resulted in cell death.

Terminal differentiation of odontoblasts requires structural integrity of the cytoskeleton. Studies have shown that the cytoskeleton of eukaryotic cells play an important role in cellular functions such as motility, mitosis, morphology and anchorage-adherent growth. At 4 h apical accumulation of actin was observed and alignment of actin was observed at 24 h inT4-4 cells on DPP substrate. Published studies have shown that colchisin, vinblastin as well as cytochalasin B were found to inhibit polarization of odontoblasts (Miake et al., 1982; Ruch et al., 1995). Thus, cytoskeleton plays an important role in odontoblast polarization. Interestingly, within 24 h, phosphorylation of focal adhesion kinase (FAK) and paxillin were observed, suggesting the importance of the focal adhesion components activated by DPP. Focal adhesions are sites of cell attachment to the extracellular matrix where transmembrane integrins link the ECM to the cytoskeleton (Tureckova et al., 2009). Published reports suggest that FAK is activated through integrin receptors and gets recruited to integrins by paxillin, vinculin, and talin (Tureckova et al., 2009). This suggests that DPP in the matrix can influence the polarized alignment of actin, probably generating a mechanical force required for activation of paxillin and FAK. Published studies have shown that uniaxial mechanical stretch can stimulate the activation of ERK, p38MAPK and Akt pathways in dental pulp stem cells (Hata et al., 2013).

This led us to investigate the role of PDK1 a master kinase and Akt as a mediator of preodontoblast differentiation. Phosphorylation of PDK1 phosphorylation at Ser241 and Akt phosphorylation at Ser 473 were observed at early time points when T4-4 cells were cultured on DPP substrate. Interestingly, Akt phosphorylation levels declined at 24 h, suggesting that Akt activation is mainly required during early events of cell adhesion and survival. The inhibitor LY294002 which inhibits activation of AKT, downregulated DPP-induced AKT activation. These findings confirm the specificity of DPP in activating the AKT signaling pathway in preodontoblasts.

A key effector of AKT-induced signaling is the regulatory protein mTOR. mTOR regulates a number of functions stimulated by Akt activation such as cell cycle, proliferation, cytoskeletal organization and cell differentiation (Hayden et al., 2006; Skeen et al., 2006; Laplante and Sabatini, 2013). Previous studies have shown that mTOR plays an important role in odontoblast differentiation (Kim et al., 2011). Our data showed an increase in mTOR expression at the specified time points when plated on DPP substrate. mTOR exists in two different conformations in mammalian cells; mTOR complex-1 (mTORC1;mTOR-Raptor complex) and mTOR complex-2 (mTORC2; mTORC2; mTOR-Rictor complex) (Laplante and Sabatini, 2013). In this study mTOR is phosphorylated on Ser 2448 suggesting mTORC2 as the predominant form. Thus, DPP in the matrix can upregulate p-AKT and p-mTOR, predominantly mTORC2 which suggests that DPP mediated activation of AKT-mTOR signaling is required for preodontoblast differentiation.

In this study, we also demonstrate that the AKT-mTOR signaling pathway can also activate NF-κB (phosphorylation of Ser 538). The NF-κB family is comprised of five closely related members: p65/RelA, c-Rel, RelB, NF-κB1/p50, and NF-κB2/p52 (Hayden et al., 2006). This transcription factor, when inactive, resides in the cytoplasm as a heterotrimeric complex comprised of p50/p52, p65 and inhibitory kappa B (IκB). Upon phosphorylation of IκB through the activation of upstream kinases, this complex is disrupted. Dissociated p-IkB is ubiquitinated and degrades, whereas the remaining heterodimeric complexes comprised of p50/p65 and p52/p65 translocate to the nucleus to perform transcriptional functions. Results show that the serine 536 of NF-κBp65 can be activated by DPP during the differentiation process.

The cell cycle is controlled by cyclins, cyclin-dependent kinases (Cdks) and cyclin-dependent kinase inhibitors. Cyclins and cdks activate cell cycle factors essential for the start of the next cycle phase. Cells passing through the the G1 phase and following S phase entry requires the activities of the D-type cyclins and the cyclin D-dependent kinases. In this study, we observed increased expression levels of cyclin D1 and Cdk4 when cells were on DPP substrate at early time points. Lower expression levels of these cell cycle factors at 16 and 24 h suggest that after the proliferation phase DPP can promote cell differentiation.

Terminal differentiation of preodontoblasts was confirmed by the synthesis of major odontoblastic ECM proteins such as DMP1, DSP, and DPP. Formation of a mineralized matrix as assessed by von Kossa and Alizarin Red staining confirmed the functional nature of the odontoblast-like cells.

**Figure 6:**
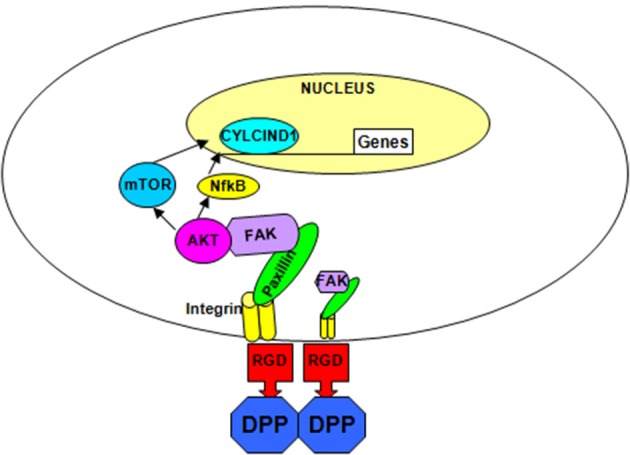
**Hypothetical model**. The hypothetical model depicts the integrin-mediated anchorage of T4-4 cells on DPP substrate and the subsequent activation of focal adhesion complexes, survival and differentiation pathway in T4-4cells.

Overall, we have shown that DPP on the matrix can activate the cytoskeletal elements resulting in increased phosphorylation of AKT, mammalian target of rapamycin and the transcription factor NF-κB leading to cell survival and increased proliferation (Figure 6). In the presence of differentiation medium, the DPP substrate could promote terminal differentiation of preodontoblasts. These data strongly suggest that DPP could be a suitable target for therapeutic interventions for the proliferation and differentiation of preodontoblasts to fully functional odontoblasts. Dental pulp cells and preodontoblasts are responsive to the DPP signal from the matrix. Therefore, for regenerating or tissue engineering dentin, scaffolds could be immobilized with DPP for facilitating proliferation of stem cells or preodontoblasts based on the AKT-mTOR –cyclin D1 signaling pathway. DPP stimulus in the presence of a mineralization microenvironment can switch the proliferating cells into an odontoblast differentiation pathway. Such a strategy could be used for regenerating damaged dentin using preodontoblasts or stem cells.

## Author contributions

AE Conceptualized, planned, performed all the experiments and wrote part of the manuscript. AG Was involved in conceptualization and planning with AE. Contributed toward writing, editing, and proofreading the manuscript along with AE.

## Funding

This project was funded by the NIH grant DE 19633 and the Brodie Endowment fund.

### Conflict of interest statement

The authors declare that the research was conducted in the absence of any commercial or financial relationships that could be construed as a potential conflict of interest.
